# Use of 3D faces facilitates facial expression recognition in children

**DOI:** 10.1038/srep45464

**Published:** 2017-04-03

**Authors:** Lamei Wang, Wenfeng Chen, Hong Li

**Affiliations:** 1 Joint International Research Laboratory of Child Development and Health, College of Psychology and Sociology, Shenzhen University, Shenzhen, China; 2 Institute of Psychology, Chinese Academy of Sciences, Beijing, China

## Abstract

This study assessed whether presenting 3D face stimuli could facilitate children’s facial expression recognition. Seventy-one children aged between 3 and 6 participated in the study. Their task was to judge whether a face presented in each trial showed a happy or fearful expression. Half of the face stimuli were shown with 3D representations, whereas the other half of the images were shown as 2D pictures. We compared expression recognition under these conditions. The results showed that the use of 3D faces improved the speed of facial expression recognition in both boys and girls. Moreover, 3D faces improved boys’ recognition accuracy for fearful expressions. Since fear is the most difficult facial expression for children to recognize, the facilitation effect of 3D faces has important practical implications for children with difficulties in facial expression recognition. The potential benefits of 3D representation for other expressions also have implications for developing more realistic assessments of children’s expression recognition.

Understanding others’ feelings from facial expressions is essential for children’s social development and adaptation^1^. Traditionally, assessments of children’s ability to recognize facial expressions have mainly relied on two-dimensional (2D) face stimuli. Little is known about how the difference between this and real faces, which are three-dimensional (3D), impacts the accuracy of these assessments.

One of the key differences between 2D face and 3D face stimuli is the availability of depth cues. In reality, direct social interactions typically occur at a distance, where stereopsis provides strong depth cues through binocular vision. Although 3D representation plays a relatively minor role in adults’ face identity recognition^2^,^3^, little is known about whether the same is true for children. Research reveals that 3D information may be useful for children’s performance in other kinds of cognitive tasks. For example, it creates a more effective mode of representation for children’s sequential time perception compared to 2D pictorial scripts^4^; teaching in a 3D environment improves children’s cognitive modifiability relative to a 2D environment^5^. Moreover, 3D virtual reality can increase the awareness of teenagers about the social and emotional experiences of their classmates^6^. These findings show that 3D representations may play an important role in children’s social-cognitive development. It is therefore sensible to investigate what differences 3D information may bring into children’s development of face perception. The main aim of the present study was to understand whether 3D representations could facilitate children’s recognition of facial expression.

Studies show that children’s discrimination of facial expression becomes progressively more accurate from early to middle childhood. Infants as young as a few months old have been shown to be able to visually discriminate among facial expressions, as indicated by eye-movement paradigms such as habituation and preferential looking^7^. There is also behavioral evidence that by 12 months of age, infants can adjust their behaviors according to their caregivers’ positive or negative facial expressions^8^,^9^. Children’s ability to verbally relate facial expressions to specific emotional states appears between 2 and 3 years of age. The first facial expressions recognized by children are happiness, followed by sadness and anger^10^,^11^,^12^, while expressions of fear, surprise, and disgust appear to be recognized later, at approximately 4 years of age^13^,^14^,^15^,^16^. Chinese children have been found to show a similar developmental trajectory of facial emotion recognition^17^. The literature thus suggests that children’s recognition of specific facial expressions develops with age in a rather universal developmental pattern, in which children first learn to recognize happy expression and later for fear.

Over the years, children’s ability to identify facial expressions has been examined in a variety of studies (for a review, see ref. 18). However, to our knowledge, all studies to date have used 2D photographs or pictures to test children’s recognition of facial expression. This limitation makes it difficult to assess the role of 3D shapes in recognition. The current study aimed to bridge this gap in the literature. We examined the hypothesis that children recognize facial expressions more accurately when face stimuli are presented in a 3D face format.

Compared to 2D face images, 3D faces retain more information about the face geometry. For example, the height of a nose in a full frontal view can only be accurately estimated when the depth cue is available. In the object recognition literature, it has been shown that stereo viewing of objects can lead to better generalization to new views^19^,^20^. Stereo viewing improves shape discrimination even when rich monocular shape cues are available^21^.

Face perception may also involve 3D shape processing that derives surface structure from available depth cues such as texture and shading^22^. In fact, compared to object recognition, face recognition appears to depend more on representations of surface properties such as shading^22^. In a 3D face model, facial features are represented by local and global curvatures that can be considered the true identifying characteristics of a person^23^. Research has shown that 3D information does at times improve face identification performance across simulated depth planes^2^ and different face views^24^,^25^.

Although these studies have shown that 3D information is useful for the recognition of face identity, it is not known whether it is also useful for the recognition of facial expressions. Given that children are less skilled than adults in recognizing facial expression, we hypothesized that children’s performance on facial expression recognition tasks could benefit more from depth cues, as a more accurate representation of facial features in 3D should lead to potentially higher discriminating power^26^.

It has been shown that, along with the participant’s age and gender, the type and intensity of facial expressions are important predictors for children’s performance in expression-processing tasks^27^,^28^. Especially the female advantage in facial expression processing has long been documented^27^. Among basic facial expressions, happiness is recognized earliest and most accurately, while fear is recognized latest and least accurately by children^29^,^30^. According to Bullock and Russell’s^31^,^32^,^33^ structural model, happiness and fear are at the two opposite sides of pleasantness. For both expressions, children’s performance in free-labeling tasks improves over the ages of 3, 4 and 5 years^34^. Thus, we chose these expressions for children to recognize in the current study.

Taking these previous findings into account, we could hypothesize that the use of 3D faces would facilitate children’s facial expression recognition in processing speed and accuracy. Further, we expect children’s facial expression processing would also be affected by children’s age, gender, expression intensity and expression category as found in children’s processing of 2D faces.

## Material and Methods

### Participants

Seventy-one children aged between 3 and 6 participated in the study. Thirty-five of them were from the first year (*M*_age = _4 years 1 month, *SD* = 2 months, 17 girls), and 36 were from the third year (*M*_age = _5 years 7 months, *SD* = 3 months, 18 girls) of a kindergarten in Beijing. All children had normal vision and no known psychiatric disorders. Informed consent was obtained from the legal guardians of all participating children. The protocol was approved by the Institutional Review Board (IRB) at the Institute of Psychology, Chinese Academy of Sciences. The methods were carried out in accordance with the Declaration of Helsinki.

### Materials

We employed 4 Chinese models (2 males and 2 females) with 2 expressions (fear and happiness) of 4 intensity levels. These faces were presented in two image formats, either 2D or 3D representations. This amounted to a total of 64 face stimuli (4 models × 2 expressions × 4 intensities × 2 image formats). Two additional models and the same conditions were used in practice trials. The face stimuli were chosen from the BU-3DFE database^35^. Yin and colleagues^35^ used the 3D face imaging system (3DMD digitizer) to merge six synchronized cameras’ viewpoints data and to produce a single 3D face surface map. The models were requested to perform each expression for a short period of time. The 4 intensity levels of the expression were captured by asking the model to perform the light (low) intensity of each expression first to simulate the spontaneity of the emotional state; then each model was requested to perform four stages of expressions, ranging from low intensity, middle, high, and highest intensity of a specific expression. It was up to the model to display four stages of expressions with his/her own style. Finally, each expression data set was validated in three steps, by the model him/herself, by experts in interpreting facial expressions, and by machines via facial expression recognizer. The final database contains 2D images, 3D models and texture maps of facial expressions from 100 persons. The emotional faces resembled real color photographs and had no facial hair or eyeglasses.

In the 2D image format condition of the current study, the 2D pictorial image was displayed. In the 3D image format condition, the 3D face model with its associated texture was rendered on the screen. The object file format of the 3D models was first converted to Open Inventor file format (http://oss.sgi.com/projects/inventor/). They were then displayed with VRVision^36^, which was specially developed for displaying 3D stimuli in Matlab. VRVision serves as an interface between the Matlab and Open Inventor graphics environments, displaying the 3D faces with graphics libraries according to the predefined image conditions. Face images were shown in full color against a neutral gray background. The face height from the top of the forehead to the tip of the chin was approximately 10 cm (about 9.5 degree of visual angle at a distance of 60 cm). The face width was about 7.6–8.6 cm (7.2–8.2 degree).

The face stimuli were displayed on a laptop with a 17-inch monitor (Lenovo, IdeaPad Y570). The screen resolution was set at 1024 × 768 with 32-bit color. The software for the experimental control was written in Matlab R2012 with Psychophysics Toolbox extensions^37^,^38^.

To confirm that the 3D information could be perceived for the 3D image format, we recruited another sample of 20 kindergarten children to rate the subjective sense of 3D on a 3-point scale (−1 meant “I think it is 2D”, and 1 meant “I think it is 3D”, and 0 means “I can’t tell”) for each of the 64 test face stimuli including both 2D and 3D image formats. Eight practice trials were given before the rating. Binomial tests of a null hypothesis (*p *<=* *0.5) about the probability of success were conducted on the rating for 2D and 3D images separately. The results showed that children didn’t rate the subjective sense of 3D for the 2D and 3D image formats randomly (*p*’s < 0.001). They appeared to be able to discriminate 2D and 3D image formats.

### Procedures

We employed a two-alternative forced-choice task, which required the children to judge whether a face in each trial showed a happy or fearful expression.

#### Practice

Two keys on the keyboard were each tagged with happy or fearful emoji labels. Prior to the task, children were asked to identify the emotion of each label. Wrong answers were corrected until they correctly identified the labels. This was followed by the two practice faces, which were shown one at a time in the center of the screen. Each face was presented once with a happy expression and once with a fearful expression, and each face was shown once in a 2D format and once in a 3D format. Children were asked to judge the expression by pressing one of the labeled keys as quickly and as accurately as possible. Wrong responses were corrected until the correct answer was chosen. The 8 practice trials (2 faces × 2 expressions × 2 image formats) were executed in a random order.

#### Test

The experimental trials followed immediately after the practice. The 2D and 3D conditions were tested in separate blocks. The order of the two blocks was counterbalanced across age and gender such that half viewed 2D faces first and the other half viewed 3D faces first. The 32 face stimuli in each block were presented in a random order. Each trial began with a 500 ms central fixation, followed by a face on the screen for 8 seconds or until a response was made. This procedure was identical to the practice trials.

## Results

First we checked the assumptions for ANOVA and confirmed that the residuals were normally distributed and the equal variances can be assumed (*p’*s > 0.05). Outliers in the reaction time were excluded on the basis of a two-standard-deviation cut. The final accuracy data (*M* = 0.72, *SD* = 0.15) and reaction time data (*M* = 2.09, *SD* = 1.47) of the 71 subjects were subjected to 5-way mixed-design ANOVAs (2 image formats × 2 expressions × 4 intensities × 2 age groups × 2 genders).

For reaction time, a main effect of image format was found (*F* (1, 67) = 4.32, *p* = 0.04, *η*^*2*^_*p*_ = 0.06). Children’s recognition of facial expression for 3D faces (*M* = 1.88 s, *SD* = 1.26) was significantly faster than that for 2D faces (*M* = 2.31 s, *SD* = 0.96). There was also a main effect of age (*F* (1, 67) = 19.74, *p < *0.001, *η*^*2*^_*p*_ = 0.23). Children in the older age group recognized facial expressions faster (*M* = 1.40 s, *SD* = 0.22) than those the younger group (*M* = 2.79 s, *SD* = 0.22). No other main effects or interaction effects were found in the reaction time data (*p*’s > 0.06).

For accuracy data, the image format effect on accuracy (*F* (1, 67) = 2.01, *p* = 0.16, *η*^*2*^_*p*_ = 0.03) was not significant. The proportion accuracies for 3D faces (*M* = 0.73, *SD* = 0.10) and 2D faces (*M* = 0.71, *SD* = 0.09) were comparable. There were significant main effects of age (*F* (1, 67) = 20.12, *p < *0.001, *η*^*2*^_*p*_ = 0.23) and intensity (*F* (3, 65) = 7.39, *p < *0.001, *η*^*2*^_*p*_ = 0.25). Performances of the older age group (*M* = 0.79, *SD* = 0.12) were more accurate than those of the younger group (*M* = 0.66, *SD* = 0.12). Accuracy improved as a function of expression intensity (*M* values for levels 1 through 4 were 0.68, 0.70, 0.74, and 0.77, respectively).

**Figure 1:**
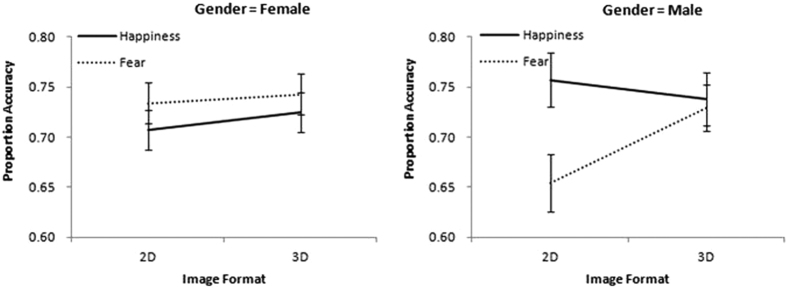
Proportion accuracy as a function of image format and expression, separately for each gender. Error bars indicate the standard error of the mean.

These main effects on accuracy were qualified by the significant interactions of image format × expression × gender (*F* (1, 67) = 5.58, *p = *0.02, *η*^*2*^_*p*_ = 0.08), as illustrated in Fig. 1. An analysis of simple effects showed that the image format × expression interaction was significant only for boys, *F* (1, 69) = 9.68, *p* < 0.001, *η*^*2*^_*p*_ = 0.30. Boys recognized fear significantly better with 3D (*M = *0.73, *SD = *0.13) than 2D (*M = *0.65, *SD = *0.18) display, while this effect was not significant for girls, *F* (1, 69) = 0.09, *p* = 0.77, *η*^*2*^_*p*_ = 0.01.

Further simple effect analysis of the three-way interaction on accuracy also revealed a significant simple interaction effects of expression × gender (*F* (1, 69) = 6.17, *p = *0.003, *η*^*2*^_*p*_ = 0.14) for 2D faces. For 2D image formats, boys recognized happiness (*M = *0.75, *SD = *0.17) equally as well as girls (*M = *0.71, *SD = *0.16), whereas they performed significantly worse for fear (*M = *0.66, *SD = *0.17) than girls (*M = *0.73, *SD = *0.17).

To evaluate the impact of the variation in the perception of 3Dness on the recognition performance, an item-based regression analysis on accuracy and reaction time with the frequency of each image rated as 3D as a prediction variable was conducted. The results revealed that the frequency of each image rated as 3D could significantly predict (*β* = 0.50, *p* < 0.001, Adjusted *R*^*2*^ = 0.24) the reaction time, but not the accuracy (*p* = 0.62), which is in line with the effect of image format in the subject-based analysis.

## Discussion

This study examined the role of 3D information in children’s recognition of facial expression. The results not only showed a significant advantage of this information in the speed of children’s recognition of facial expressions but also showed a benefit in improving boys’ recognition of fearful expressions. These findings suggest that children could recognize 3D facial expressions more efficiently and it especially benefits boys in recognizing difficult facial expressions when 3D information is available. This implies that past research based on 2D images may have underestimated children’s ability to recognize certain expressions in real life, where 3D information is available.

Previous researchers have suggested that using 3D faces could lead to better identity recognition performance (e.g., 2; 24; 25). Our results now confirm that 3D information can also lead to faster recognition of facial expressions in kindergarten children and higher accuracy for recognizing difficult emotions for boys. One cause of the facilitating effect in facial expression processing speed may be the additional depth cues that make the expressive features more identifiable. Another contributing factor may be the ecological validity of 3D representations. Children may be more familiar with 3D faces they encounter in real life. Presenting face stimuli in stereopsis resembles real faces more closely. This may be affected by a degree of encoding specificity for 2D and 3D information in face processing. As suggested in the literature, transfer between 2D and 3D representations of faces could compromise identification accuracy even in adults^3^. Matching 3D faces observed daily by children to a 2D representation on the screen requires establishing a correspondence between stereopsis and the discrepant information derived from monocular depth cues. The results thus may suggest that there is a cost of time for children to transfer information across 2D-3D representations. Consistent with prior research^27^, which showed a small but robust female advantage in facial expression recognition from infancy into adolescence, our results also showed that girls outperform boys in expression recognition. A more recent meta-analysis^39^ on emotion recognition revealed that this gender difference depends on the emotion category: larger gender differences are found for negative emotions such as fear. Girls perform especially better than boys for negative emotions. The current study confirms this finding by showing that when the faces were shown in 2D image format, boys performed less accurately than girls in recognizing fearful expressions, while they recognized happy expressions equally well as for girls. Nevertheless, when the faces were shown in 3D image format, boys’ accuracy in recognizing fearful expression has been significantly improved to the same level as girls’. Our results indicate that we may underestimate the emotional ability of boys when understanding the gender difference in development. It is well known that boys are better developed in spatial ability than girls, whereas worse in emotional ability than girls. Our results suggest the weakness in boys’ emotional ability may not be so obvious when the information provided capitalizes on their spatial information processing strengths.

Our results also confirmed that children’s understanding of specific facial expressions improved with age, that older children recognized facial expressions faster and more accurately. Although the effects of both image format and age were manifested in terms of speed and accuracy, the effects of expression category, expression intensity and children’s gender were only manifested in term of accuracy. It may be speculated that the effect of image format is robust and strong as the effect of age. An alternative explanation is that the reaction time is not a sensitive or reliable index to measure the performance of facial expressions recognition in children. Indeed, our data indicated that the extent of variability of reaction time was far larger than of accuracies in term of coefficient of variation (0.71 vs. 0.16).

Recognizing facial expressions is crucial for successful social communication and adaptation. Our finding that 3D representations improve children’s recognition of fearful expressions may apply to children with difficulties in facial expression recognition. It has been shown that higher accuracy for recognizing fearful facial expressions can predict prosocial behaviors^40^. Difficulties in recognizing facial expressions can have long-term detrimental effects on social behavior, which are linked to risk factors for maladjustment and lower social competence^41^. Children with autism or psychopathic tendencies often have difficulties in recognizing facial expressions^42^,^43^. By employing 3D techniques, researchers and caretakers may acquire better tools to train these children to recognize facial expressions in real life.

In sum, our findings suggest that, even though 2D images are most common in research on facial expression processing, it may be beneficial to start employing 3D methods so that children’s development in expression recognition can be evaluated more accurately. A 3D approach may also be useful for more effectively training children with expression recognition deficits.

## Additional Information

**How to cite this article**: Wang, L. *et al*. Use of 3D faces facilitates facial expression recognition in children. *Sci. Rep.* **7**, 45464; doi: 10.1038/srep45464 (2017).

**Publisher's note:** Springer Nature remains neutral with regard to jurisdictional claims in published maps and institutional affiliations.
